# Neutrophil‐Monocyte‐to‐Lymphocyte Ratio as a Valid Prognostic Tool for Acute Ischemic Stroke With Performance Comparable to Neutrophil‐to‐Lymphocyte Ratio

**DOI:** 10.1002/brb3.71426

**Published:** 2026-04-21

**Authors:** Yuan Zhao, Li Yi, Shen Shen, Liu Yang, Hongmin Li, Beibei Liu, Yongbo Zhang

**Affiliations:** ^1^ Department of Neurology Beijing Friendship Hospital, Capital Medical University Beijing China

**Keywords:** acute ischemic stroke, inflammation, neutrophil‐monocyte‐to‐lymphocyte ratio (NMLR), prognosis

## Abstract

**Background:**

Inflammation is a key mediator for brain injury in acute ischemic stroke (AIS) and the neutrophil‐monocyte‐to‐lymphocyte ratio (NMLR) is a novel composite index for inflammatory. So far, prior studies have not evaluated the prognostic value of NMLR for 3‐month functional outcomes in patients with AIS, nor systematically compared its performance with established markers, such as neutrophil‐to‐lymphocyte ratio (NLR), monocyte‐to‐lymphocyte ratio (MLR), and individual cellular components. This prospective cohort study aims to address these research gaps.

**Methods:**

A total of 815 consecutive patients with AIS hospitalized between December 2023 and May 2024 were enrolled. Baseline clinical data and blood samples were collected within 24 h of admission. The primary outcome was poor 90‐day functional outcome (modified Rankin Scale score > 2). Multivariable logistic regression determined adjusted odds ratios (aORs) and 95% confidence intervals (CIs) for the outcome association of NMLR. Receiver operating characteristic (ROC) curve analysis compared the predictive performance (area under the curve [AUC]) of NMLR with NLR, MLR, and individual cellular components.

**Results:**

Of the 815 patients, 224 (27.5%) had poor functional outcomes. After adjusting for confounders, higher NMLR was independently associated with increased risk of poor outcomes (aOR = 1.370 per unit increase, 95% CI: 1.214–1.546; *p* < 0.001). NMLR's predictive performance (AUC = 0.708, 95% CI: 0.668–0.749) was statistically equivalent to NLR (AUC = 0.709, *p* = 0.594), but significantly superior to MLR and individual cellular components (all *p* < 0.05).

**Conclusions:**

This is the first prospective study to assess the predictive value of the NMLR for 3‐month functional outcomes in patients with AIS and systematically compare NMLR with NLR. NMLR can predict poor 90‐day outcomes in patients with AIS independently. NMLR is a valid prognostic tool for AIS with performance comparable to NLR.

## Graphical abstract

The neutrophil‐monocyte‐to‐lymphocyte ratio is a valid prognostic tool for acute ischemic stroke with performance comparable to the neutrophil‐to‐lymphocyte ratio.

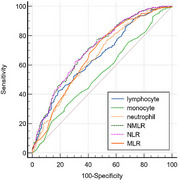

## 1 Introduction

Stroke remains a major public health challenge in China. The national representative data in 2020 show that the prevalence rate of stroke is 2.6%, and the incidence rate is 505.2 cases per 100,000 people per year among adults aged 40 years or older, which highlights the huge disease burden and requires more accurate prognosis assessment (Tu et al. 2023). The concept of “thrombo‐inflammation” underscores the critical interplay between inflammatory and thrombotic pathways in driving brain injury after acute ischemic stroke (AIS) (Stoll and Nieswandt 2019). Consequently, readily available composite indices derived from routine blood counts have emerged as practical prognostic tools, correlating with stroke severity and outcomes (Zhang et al. 2021; Sarejloo et al. 2022; Zhai et al. 2022).

To advance this field, we prospectively validated a novel integrative index, that is, the neutrophil‐monocyte‐to‐lymphocyte ratio (NMLR) and evaluated its prognostic utility. The NMLR reflects three interlinked pathophysiological dimensions: the acute neutrophilic response (Chen et al. 2025), lymphocytic immunoregulation (N. Li et al. 2025), and monocyte‐mediated processes (Lyu et al. 2021). The NMLR allows a more holistic assessment of the poststroke “inflammation‐immunity‐repair” axis. However, the independent prognostic value of the NMLR in AIS and its potential advantage over the established neutrophil‐to‐lymphocyte ratio (NLR) remain undetermined.

We hypothesized that the NMLR could provide superior prognostic value. Therefrom, this prospective cohort study had two principal objectives: first, to determine if admission NMLR independently predicts 3‐month poor functional outcome; second, to compare the predictive performance of NMLR with NLR.

## 2 Methods

### 2.1 Study Design

This prospective, single‐center cohort study was conducted at the Department of Neurology, Beijing Friendship Hospital, Capital Medical University, from December 2023 to May 2024. The study was approved by the hospital's institutional review board, and written informed consent was obtained from all participants or their legal representatives. Based on a pre‐study pilot, the required sample size was calculated as 700 to achieve 90% power to detect an odds ratio of 1.3 (*α* = 0.05, two‐sided). Accounting for a 10% potential loss to follow‐up, the target enrollment was at least 800 patients.

### 2.2 Participants

**FIGURE 1:**
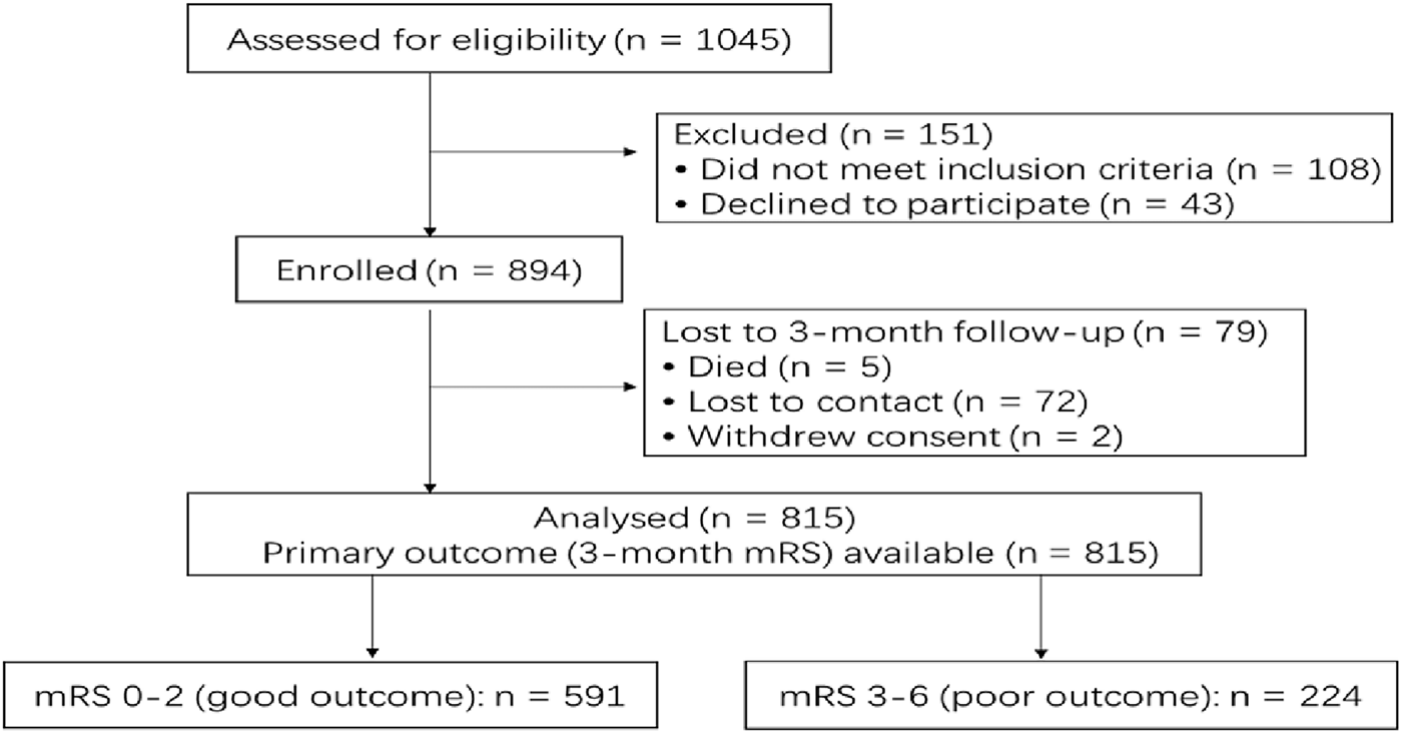
Enrollment process for patients with AIS.

A total of 1045 consecutive adults with suspected AIS were screened. Inclusion criteria were as follows: (1) age ≥ 18 years, (2) MRI‐confirmed AIS, and (3) admission within 72 h of symptom onset. Exclusion criteria were as follows: (1) history of malignancy or severe hepatic/renal dysfunction, (2) active infection or systemic inflammatory disease within 4 weeks prior to admission, or (3) missing baseline laboratory data. After applying these criteria, 815 patients were included in the final analysis cohort (**Figure** **1**).

### 2.3 Procedures

Baseline demographic, clinical, and laboratory data were collected upon admission. Fasting venous blood was drawn within 24 h. Collected variables included demographics, medical history, stroke severity (National Institutes of Health Stroke Scale [NIHSS] score), and laboratory parameters (e.g., complete blood count, C‐reactive protein [CRP]). The NMLR was calculated as (neutrophil count + monocyte count)/lymphocyte count.

Stroke etiology was classified using the Trial of Org 10172 in Acute Stroke Treatment (TOAST) criteria (Adams et al. 1993). Cardioembolic sources were assessed via electrocardiography, 24‐h Holter monitoring, and echocardiography as clinically indicated.

The primary outcome was functional status at 3 months, assessed via telephone using the modified Rankin Scale (mRS) (Kasner 2006, Schlemm and Schlemm 2018), and dichotomized as good (scores 0–2) and poor (scores 3–6). Neuroimaging and mRS assessments were independently reviewed by two neurologists blinded to clinical data; discrepancies were resolved by consensus or a third senior neurologist, with excellent inter‐rater agreement (Cohen's kappa ≥ 0.8).

All patients received standard management per the Chinese Guidelines for Acute Ischemic Stroke (2023) (L. Liu et al. 2023), including antiplatelet therapy, statins, and vascular risk factor control.

### 2.4 Statistical Analysis

Univariable logistic regression analysis was first performed to assess the association between each variable and the outcome. Variables for the multivariable analysis were selected based on clinical relevance and results from the univariable analysis (*p* < 0.10). The final multivariable model was established using backward stepwise regression (retention criterion: *p* < 0.05), with continuous variables checked for linearity using restricted cubic splines and multicollinearity assessed (variance inflation factor [VIF] < 5). Results are presented as adjusted odds ratios (aORs) with 95% confidence intervals (CIs).

The discriminative ability of NMLR was evaluated using the area under the receiver operating characteristic (ROC) curve (AUC), with pairwise comparisons performed using the DeLong test. A two‐sided *p*‐value < 0.05 was considered statistically significant. Given that the missing data were minimal and non‐informative, a complete‐case analysis was applied. All statistical analyses were conducted using IBM SPSS Statistics 26.0.

## 3 Results

A total of 815 patients with AIS were included in this study. At the 3‐month follow‐up, 591 patients (72.5%) had a good functional outcome (mRS score 0–2), while 224 patients (27.5%) had a poor outcome (mRS score 3–6). Compared with the good‐outcome group, patients with poor outcomes were significantly older, had higher baseline NIHSS scores, longer hospital stays, and exhibited a more pronounced systemic inflammatory state, as evidenced by significantly elevated levels of NMLR and other inflammatory markers (all *p* < 0.05; **Table** **S1**). White blood cell count, neutrophil count, monocyte count, CRP, and high‐sensitivity CRP all showed a significant and progressively increasing trend across ascending NMLR quartiles. Conversely, lymphocyte count gradually decreased with increasing NMLR quartiles (all *p* for trend < 0.05; see Table S1).

Table 1 presents the results of univariate and multivariable logistic regression analyses for poor functional outcome. After adjusting for age, history of stroke, baseline NIHSS score, length of hospital stay, hemoglobin, and HbA1c, each 1‐unit increase in NMLR remained independently associated with a 37% higher likelihood of poor outcome (aOR 1.370, 95% CI 1.214–1.546, *p* < 0.001). Furthermore, when NMLR was analyzed as a categorical variable, a significant dose‐response relationship was observed. Compared with the lowest quartile (Q1), the adjusted odds of a poor outcome were nearly tripled in Q3 (aOR 2.779, 95% CI 1.650–4.680) and nearly sixfold higher in the highest quartile (Q4; aOR 5.793, 95% CI 3.457–9.709; *p* for trend < 0.001). Tests for nonlinearity using restricted cubic splines confirmed a linear relationship for all continuous variables in the final model (all *p* for nonlinearity > 0.05). Multicollinearity was assessed using the VIF, and all variables in the final model had a VIF of less than 5, indicating no significant multicollinearity.​

**TABLE 1:** Univariate and multivariable logistic regression analysis of factors associated with poor 3‐month functional outcome.

Variable	Univariate analysis		Multivariable analysis
	OR (95% CI)​	*p* value​	Adjusted OR (95% CI)​
Demographics​			
Age	1.039 (1.024–1.055)	< 0.001	1.036 (1.013–1.060)
Stroke risk factors​			
History of stroke	0.446 (0.316–0.630)	< 0.001	0.542 (0.334–0.878)
Clinical parameters​			
NIHSS score at admission	1.963 (1.764–2.185)	< 0.001	1.993 (1.723–2.168)
Hospital stay	1.076 (1.039–1.114)	< 0.001	1.046 (1.016–1.076)
Laboratory parameters​			
Hemoglobin	0.984 (0.975–0.993)	0.001	0.987 (0.973–1.000)
HbA1c	1.097 (1.037–1.160)	< 0.001	1.105 (1.012–1.207)
NMLR​			
NMLR	1.498 (1.361–1.648)	< 0.001	1.370 (1.214–1.546)
NMLR quartiles (vs. Q1)			
Q1 (≤ 2.011)	1.00 (Reference)		1.00 (Reference)
Q2 (2.012–2.762)	1.599 (0.939–2.723)	0.084	1.588 (0.971–2.750)
Q3 (2.763–3.782)	2.731 (1.649–4.524)	< 0.001	2.779 (1.650–4.680)
Q4 (> 3.782)	5.999 (3.676–9.789)	< 0.001	5.793 (3.457–9.709)
p for trend​	< 0.001		< 0.001

*Note*: The multivariable model was adjusted for all variables listed in the table (age, history of stroke, NIHSS score, hospital stay, hemoglobin, and HbA1c).

Abbreviations: CI, confidence interval; HbA1c, Hemoglobin A1c; NIHSS, National Institutes of Health Stroke Scale; NMLR, neutrophil‐monocyte‐to‐lymphocyte ratio; OR, odds ratio.

**FIGURE 2:**
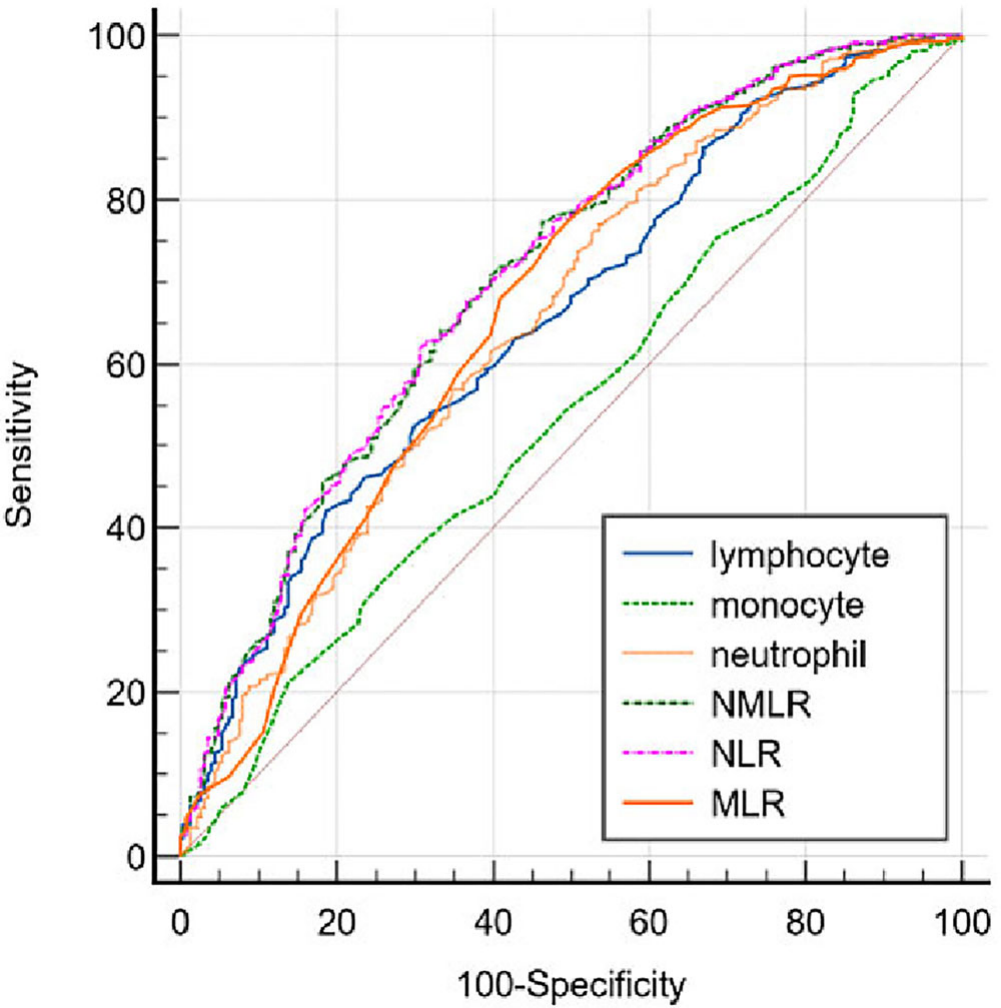
ROC curve for predicting the prognosis of AIS at 3 months using inflammatory markers. ROC curves for the NMLR, NLR, MLR, and their cellular components in predicting poor 3‐month functional outcome. The corresponding AUC values, detailed in Table 2, demonstrate that the NMLR (AUC = 0.708) and NLR (AUC = 0.709) have equivalent predictive performance (*p* = 0.594) and that both composite ratios outperform the MLR and all individual cell counts.

Table 2 summarizes the discriminative performance of NMLR and other inflammatory indices. The NMLR demonstrated an AUC of 0.708 (95% CI 0.668–0.749) for predicting poor functional outcome, with statistically equivalent performance to the NLR (AUC 0.709, *p* for comparison = 0.594). Both NMLR and NLR significantly outperformed the monocyte‐to‐lymphocyte ratio (MLR) and all individual cellular components in discriminative ability (all *p* < 0.05). Using the Youden index to determine the optimal cutoff point, an admission NMLR value of 3.2 was identified, corresponding to a sensitivity of 70.7% and a specificity of 60.3%. The corresponding ROC curves are shown in Figure 2.

**TABLE 2:** Predictive performance of inflammatory indices for poor 3‐month functional outcome.

Indicator	AUC (95% CI)	Sensitivity, %	Specificity, %	Youden index, %	AUC difference versus NMLR (95% CI)	p value
NMLR​	0.708 (0.668–0.749)	70.7	60.3	31.0	—(Reference)	—
NLR	0.709 (0.669–0.750)	62.1	69.2	31.3	0.001 (−0.002 to 0.004)	0.594
MLR	0.668 (0.624–0.712)	77.8	50.0	27.8	0.040 (0.008–0.072)	0.013​
Neutrophil count (×10^9^/L)	0.652 (0.609–0.696)	47.3	76.1	23.4	0.056 (0.022–0.090)	0.002​
Lymphocyte count (×10^9^/L)	0.656 (0.614–0.698)	61.6	61.8	23.4	0.052 (0.021–0.084)	0.001​
Monocyte count (×10^9^/L)	0.544 (0.499–0.588)	74.1	33.5	7.6	0.165 (0.110–0.219)	< 0.001​

*Note*: Pairwise comparisons of the AUC for each indicator against the NMLR (reference) were performed using the DeLong test.

Abbreviations**: AU**C, area under the receiver operating characteristic curve; CI, confidence interval; MLR, monocyte‐to‐lymphocyte ratio; NLR, neutrophil‐to‐lymphocyte ratio; NMLR, neutrophil‐monocyte‐to‐lymphocyte ratio.

## 4 Discussion

To our knowledge, this is the first prospective study to assess the predictive value of the NMLR for 3‐month functional outcomes in patients with AIS and systematically compare NMLR with the NLR. Our findings confirm that NMLR can predict poor 3‐month AIS outcomes independently, with prognostic performance comparable to the well‐established NLR, and NMLR was statistically equivalent to NLR, but significantly superior to MLR and individual cellular components.

Previous studies have demonstrated that the NLR is an independent risk factor for AIS (Kocaturk et al. 2019; L. Li et al. 2021; Qiu et al. 2023; Y. Liu et al. 2022). Although our study found that NMLR and NLR had similar efficacy in predicting 3‐month poor functional outcomes in patients with AIS (AUC 0.708 vs. 0.709, respectively), this does not mean that NMLR is redundant. On the contrary, this result showed the high efficiency of NMLR as a novel composite inflammatory indicator. NMLR achieves an assessment of poststroke immune inflammation by incorporating monocytes into the calculation. First, NMLR covers three core immune cells, including neutrophils, lymphocytes, and monocytes. Neutrophils are the main executors of acute inflammatory injury (Kang et al. 2020). Neutrophils directly destroy the blood‐brain barrier and cause cell death by releasing reactive oxygen species (ROS), matrix metalloproteinases (MMPs), neutrophil extracellular traps (NETs), etc (Xie et al. 2023). Lymphocytes serve as a pivotal hub for immune regulation and neural repair (N. Li et al. 2025). Lymphopenia serves as a marker for both immunosuppression and reduced repair potential after AIS (Wang, Ye, et al. 2022). Monocytes differentiate into macrophages in tissues, and the M1/M2 polarization balance of macrophages affects tissue clearance, inflammation resolution, and repair processes after AIS (Lyu et al. 2021; Wang, Pan, et al. 2022; Ley et al. 2011). Therefore, NMLR integrates three core immune cells and reflects the immune imbalance after AIS. Second, monocytes are the main source of foam cells in atherosclerotic plaques and are directly related to the formation and rupture of vulnerable plaques (Kim et al. 2020). Therefore, NMLR may be more suitable for the prognostic evaluation of large artery atherosclerotic stroke. This is a dimension that NLR lacks. Third, by correlating the repair function of macrophages, NMLR provides clues about the long‐term potential of nerve repair, and its predictive value may be related to the long‐term prognosis after AIS. The NMLR captures information about poststroke “thrombosis and inflammation”. In contrast, the NLR merely reflects the neutrophil‐to‐lymphocyte ratio and does not account for key monocyte‐driven inflammatory and repair processes. Therefore, our study confirms that NMLR is equivalent to NLR in prediction efficiency and verifies its reliability as a prognostic tool. More importantly, NMLR integrates more immune cells, providing a theoretical basis for predicting the prognosis of AIS.

Our study is an enhancement and expansion based on previous evidence. Previous studies have suggested that NMLR has predictive value in​ patients with acute myocardial infarction (Yan et al. 2020; Wang, Yuan, et al. 2022) and in those with AIS after thrombectomy (Gao et al. 2024). Based on the above studies, our study provides​ evidence that NMLR is an independent predictor of outcomes for patients with AIS, as demonstrated by a rigorous design (prospective, consecutive enrollment, multivariate adjustment), a large sample size (*n* = 815), and comprehensive analyses (univariate/multivariate regression, dose‐response relationship, ROC curve comparison). We not only confirmed the association between NMLR and 3‐month prognosis in AIS patients, but also, through the dose‐response relationship (Q4 vs. Q1, OR = 5.793), demonstrated a significantly increased risk of poor prognosis with higher NMLR levels. Furthermore, ROC analysis proved that the predictive efficacy of NMLR is equivalent to that of NLR.

This study has limitations: its single‐center design restricts generalizability (needing external multicenter validation); residual confounding cannot be excluded; NMLR only reflects quantitative immune cell shifts (not functional phenotypes, e.g., activation status‐combining with flow cytometry/transcriptomics could deepen insights); and only baseline NMLR is analyzed, without assessment of dynamic changes (serial measurements may uncover additional prognostic value).

NMLR provides a calculation method, that is, (neutrophils + monocytes)/lymphocytes. Given that absolute neutrophil counts are typically an order of magnitude higher than monocyte counts in peripheral blood (as evidenced in Table S1), the numerator in NMLR is overwhelmingly dominated by the neutrophil count. Consequently, the NMLR value is primarily determined by neutrophils/lymphocytes, making it mathematically very similar to NLR. This likely explains the lack of a significant performance difference. The contribution of monocytes, as reflected in the MLR, may be mathematically “diluted” or masked in this additive formula.

In future studies, timing of blood sampling may be adjusted. In this study, blood samples were collected within 24 h of admission, but patients were enrolled within 72 h of symptom onset. This is a limitation. The inflammatory response post‐AIS is highly dynamic and time‐sensitive. Early (< 24 h) and later (24–72 h) phases may involve different inflammatory cascades and cell type kinetics. Sampling within a 72‐h window introduces substantial heterogeneity. More importantly, later time points (e.g., > 48 h) are more susceptible to the confounding influence of poststroke complications, such as infections (e.g., pneumonia), which are common and drastically alter leukocyte profiles. This confounding factor could obscure the true relationship between the stroke‐induced inflammatory response and outcome. The analysis would be strengthened by a subgroup analysis of patients sampled within a narrower, earlier window (e.g., ≤ 24 h from onset).

Moreover, the generally lower NIHSS scores may be adjusted in the future. In our study, the median baseline NIHSS score in the poor outcome group was 5 (IQR 3–8). This indicates that the cohort overall had relatively mild to moderate stroke severity. It is possible that the discriminatory power of inflammatory indices, and any potential differences between them, might be more pronounced in a population with more severe strokes. The generally lower NIHSS scores may have attenuated the ability to detect a statistically or clinically relevant difference between NMLR and NLR. This should be acknowledged as a potential factor limiting the generalizability of the findings to all AIS populations.

In conclusion, elevated admission NMLR is an independent predictor for poor 3‐month AIS outcome, with prognostic performance comparable to NLR. Unlike NLR, NMLR incorporates three key immune subsets, which include neutrophils, monocytes, and lymphocytes, thus captures the synergistic pro‐inflammatory and anti‐inflammatory responses mediated by neutrophils, monocytes, and lymphocytes. NMLR is superior to MLR and single‐cell composition as an indicator for predicting the 3‐month prognosis of AIS. Furthermore, NMLR is a valid prognostic tool for AIS with performance comparable to NLR, and its potential theoretical/comprehensive nature warrants further investigation.

## Author Contributions

**Yuan Zhao**: writing – original draft, investigation, data curation, methodology, conceptualization. **Shen Shen**: data curation, methodology. **Liu Yang**: data curation, methodology. **Hongmin Li**: data curation, methodology, conceptualization. **Yongbo Zhang**: funding acquisition, writing – review and editing, supervision. **Beibei Liu**: methodology, data curation. **Li Yi**: supervision, writing – review and editing.

## Funding

This work was supported by the National Natural Science Foundation of China (81671191, 81371355) and the Beijing Natural Science Foundation (7082028).

## Ethics Statement

This prospective study was conducted in accordance with the ethical principles of the Declaration of Helsinki. The study protocol was approved by the Ethics Committee of Beijing Friendship Hospital (2023‐P2‐284). Written informed consent was obtained from all participating patients or their legally authorized representatives.

## Consent

Informed consent was obtained from every participant.

## Conflicts of Interest

The authors declare no conflicts of interest.

## Supporting information



Supplementary Table S1. Baseline characteristics of the study population stratified by 3‐month functional outcome and NMLR quartiles.

## Data Availability

The data sets used during the current study would be available from the corresponding author upon reasonable request.
