# Supplementary material for: Neutrophil‐Monocyte‐to‐Lymphocyte Ratio as a Valid Prognostic Tool for Acute Ischemic Stroke With Performance Comparable to Neutrophil‐to‐Lymphocyte Ratio
Source: Brain Behav. 2026 Apr 21;16(4):e71426. doi: 10.1002/brb3.71426 (PMC13100358; doi:10.1002/brb3.71426)
Supplement: Supplementary file 1 — Supplementary Table S1. Baseline characteristics of the study population stratified by 3‐month functional outcome and NMLR quartiles. [file BRB3-16-e71426-s001.docx]

**Supplementary Table S1.** Baseline Characteristics Stratified by 3-Month Outcome and NMLR Quartiles in 815 Patients with Acute Ischemic Stroke

| **Characteristic** | **Functional Outcome** | |  | **NMLR Quartiles** | | | |  |
| --- | --- | --- | --- | --- | --- | --- | --- | --- |
|  | **Good (n=591)**​ | **Poor (n=224)**​ | **P Value*** | **Q1 (n=204)**​ | **Q2 (n=204)**​ | **Q3 (n=204)**​ | **Q4 (n=203)**​ | **P for Trend†**​ |
| **Demographics**​ |  |  |  |  |  |  |  |  |
| Age, years, median (IQR) | 64.0 (58.0, 70.0) | 69.0 (61.0, 77.0) | <0.001 | 65.0 (58.0, 70.0) | 64.0 (58.0, 70.0) | 64.0 (58.0, 72.0) | 69.0 (62.0, 78.0) | <0.001 |
| Male sex, n (%) | 404 (68.4) | 141 (62.9) | 0.143 | 120 (58.8) | 147 (72.1) | 137 (67.2) | 141 (69.5) | 0.028 |
| **Stroke Risk Factors, n (%)**​ |  |  |  |  |  |  |  |  |
| Hypertension | 453 (76.6) | 178 (79.5) | 0.167 | 155 (76.0) | 159 (78.0) | 155 (76.0) | 162 (79.8) | 0.620 |
| Diabetes | 293 (49.6) | 132 (58.9) | 0.050 | 100 (49.0) | 103 (50.5) | 116 (56.9) | 106 (52.2) | 0.760 |
| Hyperlipidemia | 252 (42.6) | 102 (45.5) | 0.456 | 100 (49.0) | 83 (40.7) | 90 (44.1) | 81 (39.9) | 0.232 |
| Atrial fibrillation | 36 (6.1) | 26 (11.6) | 0.008 | 10 (4.9) | 10 (4.9) | 16 (7.8) | 26 (12.8) | 0.007 |
| Coronary heart disease | 98 (16.6) | 54 (24.1) | 0.042 | 30 (14.7) | 31 (15.2) | 43 (21.1) | 48 (23.6) | 0.100 |
| Smoking history | 327 (55.3) | 113 (50.4) | 0.212 | 106 (52.0) | 107 (52.5) | 115 (56.4) | 112 (55.2) | 0.774 |
| History of stroke | 112 (19.0) | 77 (34.4) | <0.001 | 39 (19.1) | 51 (25.0) | 44 (21.6) | 55 (27.1) | 0.230 |
| **Clinical Parameters**​ |  |  |  |  |  |  |  |  |
| NIHSS score at admission, median (IQR) | 1 (0, 2) | 5 (3, 8) | <0.001 | 1 (1, 3) | 2 (1, 3) | 2 (1, 3) | 3 (1, 5.5) | <0.001 |
| Hospital stay, days, median (IQR) | 12 (10, 14) | 13 (11, 15) | <0.001 | 12 (10, 14) | 12 (10, 14) | 12 (10.5, 14) | 13 (11, 15) | 0.044 |
| **Etiology of AIS, n (%)**​ |  |  |  |  |  |  |  |  |
| Large-artery atherosclerosis (LAA) | 340 (57.5) | 135 (60.3) | 0.479 | 118 (57.8) | 108 (52.9) | 119 (58.3) | 130 (64.0) | 0.159 |
| Cardioembolism (CE) | 41 (6.9) | 31 (13.8) | 0.002 | 12 (5.9) | 17 (8.3) | 20 (9.8) | 23 (11.3) | 0.253 |
| Small-vessel occlusion (SVO) | 175 (29.6) | 52 (23.2) | 0.069 | 65 (31.9) | 68 (33.3) | 54 (26.5) | 40 (19.7) | 0.009 |
| Other/Undetermined etiology (ODE/UDE) | 35 (5.9) | 6 (2.7) | 0.059 | 9 (4.4) | 11 (5.4) | 11 (5.4) | 10 (4.9) | 0.964 |
| **Infarct Location, n (%)**​ |  |  |  |  |  |  |  |  |
| Anterior circulation infarction (ACCI) | 311 (52.6) | 126 (56.3) | 0.354 | 116 (56.9) | 107 (52.5) | 109 (53.4) | 105 (51.7) | 0.736 |
| Posterior circulation infarction (PCCI) | 228 (38.6) | 82 (36.6) | 0.605 | 75 (36.8) | 81 (39.7) | 77 (37.7) | 77 (37.9) | 0.942 |
| Mixed (both territories) | 52 (8.8) | 16 (7.1) | 0.445 | 13 (6.4) | 16 (7.8) | 18 (8.8) | 21 (10.3) | 0.527 |
| **Laboratory Parameters, median (IQR)**​ |  |  |  |  |  |  |  |  |
| White blood cell count (×10⁹/L) | 6.55 (5.47, 7.74) | 7.17 (5.98, 8.81) | <0.001 | 5.81 (4.99, 7.03) | 6.71 (5.68, 7.59) | 6.62 (5.57, 7.87) | 7.92 (6.39, 9.68) | <0.001 |
| Neutrophil count (×10⁹/L) | 4.13 (3.32, 5.04) | 4.89 (3.93, 6.35) | <0.001 | 3.16 (2.66, 3.85) | 4.21 (3.52, 4.74) | 4.58 (3.80, 5.38) | 6.02 (4.81, 7.53) | <0.001 |
| Lymphocyte count (×10⁹/L) | 1.77 (1.40, 2.18) | 1.51 (1.07, 1.84) | <0.001 | 2.14 (1.83, 2.56) | 1.92 (1.62, 2.23) | 1.54 (1.34, 1.84) | 1.19 (0.93, 1.46) | <0.001 |
| Monocyte count (×10⁹/L) | 0.39 (0.31, 0.47) | 0.41 (0.34, 0.49) | 0.053 | 0.36 (0.29, 0.43) | 0.40 (0.33, 0.48) | 0.41 (0.35, 0.49) | 0.41 (0.34, 0.54) | <0.001 |
| Red blood cell count (×10¹²/L) | 4.60 (4.27, 4.95) | 4.50 (4.09, 4.84) | 0.003 | 4.55 (4.24, 4.88) | 4.64 (4.27, 4.91) | 4.60 (4.24, 4.95) | 4.52 (4.11, 4.92) | 0.276 |
| Hemoglobin (g/L) | 142 (131, 153) | 137 (127, 148) | <0.001 | 140 (128, 150) | 142 (131, 151) | 143 (132, 154) | 138 (127, 150) | 0.021 |
| Platelet count (×10⁹/L) | 213 (181, 253) | 210 (176, 248) | 0.384 | 206 (176, 241) | 211 (179, 255) | 214 (183, 245) | 214 (177, 258) | 0.728 |
| C-reactive protein (mg/L) | 1.70 (0.83, 3.50) | 4.12 (1.33, 10.60) | <0.001 | 1.43 (0.70, 3.47) | 1.75 (0.90, 3.38) | 2.14 (1.02, 4.61) | 3.63 (1.38, 17.05) | <0.001 |
| High-sensitivity CRP (mg/L) | 1.00 (0.46, 3.26) | 3.97 (0.82, 17.09) | <0.001 | 0.81 (0.41, 2.19) | 1.08 (0.46, 3.38) | 1.54 (0.56, 4.76) | 4.65 (0.74, 24.13) | <0.001 |
| Albumin (g/L) | 39.10 (36.80, 41.70) | 38.15 (34.90, 40.28) | <0.001 | 39.30 (36.95, 41.60) | 38.95 (36.80, 41.00) | 38.70 (36.70, 41.60) | 37.80 (35.30, 41.20) | 0.007 |
| Creatinine (μmol/L) | 71.80 (61.00, 85.00) | 71.80 (59.60, 86.50) | 0.856 | 68.70 (59.25, 81.65) | 72.30 (61.20, 83.45) | 72.00 (59.30, 83.50) | 74.50 (62.00, 93.80) | 0.007 |
| HbA1c (%) | 5.80 (5.12, 7.55) | 6.17 (5.31, 8.38) | 0.002 | 5.60 (5.08, 7.19) | 5.83 (5.14, 7.71) | 6.19 (5.37, 7.90) | 5.92 (5.21, 8.19) | 0.010 |
| LDL cholesterol (mmol/L) | 2.42 (1.95, 2.99) | 2.39 (1.96, 3.06) | 0.732 | 2.56 (2.08, 3.12) | 2.41 (1.93, 2.94) | 2.37 (1.87, 2.94) | 2.40 (1.96, 3.04) | 0.039 |
| HDL cholesterol (mmol/L) | 0.97 (0.84, 1.15) | 0.99 (0.86, 1.15) | 0.720 | 0.97 (0.86, 1.18) | 0.96 (0.85, 1.14) | 0.95 (0.83, 1.12) | 0.99 (0.85, 1.16) | 0.470 |
| Homocysteine (μmol/L) | 13.30 (10.50, 18.43) | 12.95 (10.45, 19.08) | 0.614 | 12.80 (10.60, 16.90) | 13.70 (10.75, 18.90) | 13.00 (10.40, 16.60) | 13.70 (10.20, 20.50) | 0.439 |
| **NMLR**​ | 2.53 (1.90, 3.35) | 3.50 (2.55, 5.55) | <0.001 | ≤2.011 | 2.012-2.762 | 2.763-3.782 | >3.782 |  |

**NMLR Quartile Ranges**​ ≤2.011 | 2.012-2.762 | 2.763-3.782 | >3.782 |

**Footnotes:**

*P values for functional outcome groups were calculated using χ² test for categorical variables and Mann-Whitney U test for continuous variables.

†P for trend across NMLR quartiles was calculated using Mantel-Haenszel χ² test for categorical variables and linear regression for continuous variables with NMLR quartiles as an ordinal variable.

Abbreviations: IQR, interquartile range; NMLR, neutrophil-monocyte-to-lymphocyte ratio; NIHSS, National Institutes of Health Stroke Scale; CRP, C-reactive protein; LDL, low-density lipoprotein; HDL, high-density lipoprotein.
